# Will Job Crafters Stay or Leave? The Roles of Organizational Instrumentality and Inclusive Leadership

**DOI:** 10.3389/fpsyg.2021.743828

**Published:** 2021-10-08

**Authors:** Xun Xin, Wenjing Cai, Xueyuan Gao, Tingting Liu

**Affiliations:** ^1^Business School, Southwest University of Political Science and Law, Chongqing, China; ^2^Intellectual Property Research Institute, University of Science and Technology of China, Hefei, China; ^3^School of Public Affairs, University of Science and Technology of China, Hefei, China; ^4^Department of Management and Organization, Vrije University Amsterdam, Amsterdam, Netherlands; ^5^Department of Economics and Management, China University of Labor Relations, Beijing, China

**Keywords:** job crafting, organizational instrumentality, inclusive leadership, turnover intention, goal facilitation theory

## Abstract

Although studies have indicated the influences of job crafting on contemporary employees’ working outcomes, the path from job crafting to turnover intention is still unexplored in depth. Drawing on goal facilitation theory, we delineate how job crafting relates to turnover intention through organizational instrumentality and is conditioned by inclusive leadership. We collected data from 218 employees from Chinese high-tech companies at two different time points by submitting survey questionnaires. The results indicated that employees’ job crafting relates positively to their perception of organizational instrumentality and further results in decreased turnover intention. We also found that inclusive leadership not only positively moderates the path from job crafting to organizational instrumentality but also positively moderates the whole mediational relationship. Moreover, job crafting relates positively and directly to turnover intention—i.e., the more employees craft their jobs, the more likely they leave their organizations when we control the roles of organizational instrumentality and inclusive leadership. Finally, the theoretical and practical implications are also discussed.

## Introduction

With the aim of pursuing “an intelligent career” (Guan et al., 2019), contemporary employees change jobs frequently, making turnover an important issue in the management literature (Price, 2001; Waldman et al., 2015). Especially with the rapid development of the “Internet+” mode in China, many new business formats have emerged, which are bringing significant income and employment opportunities to many tech industries and their employees. Thus, employees, especially in high-tech industries, have a higher level of activity than ever before and tend to leave an organization rather than passively adapt to unsatisfactory work conditions, therefore resulting in a relatively high employee turnover rate. According to the “2017 Resignation and Salary Adjustment Research Report,” released by a leading human resources service provider in China (NASDAQ: jobs), the high-tech industry has a relatively high turnover rate of 21.6%. Employees’ voluntary turnover inevitably brings about certain losses for enterprises and affects their competitiveness (Peterson and Luthans, 2006; Park and Shaw, 2013). As the “precursor” of turnover behavior, turnover intention can effectively contribute to individual job change behavior (Cho and Lewis, 2012).

Despite substantial research on antecedents of turnover intention, whether employees who show a great deal of proactivity in the workplace are more willing to leave their jobs is still an intriguing question. Job crafting is recognized as a kind of proactive behavior that captures the idea that individuals proactively shape their jobs in terms of task, relational and cognitive aspects to align their jobs more with personal needs and work values (Wrzesniewski and Dutton, 2001; Lu et al., 2014). Over the past two decades, numerous studies have consistently found that job crafting could generate desirable job outcomes. However, we reviewed prior studies and found that the scant research to date examining how job crafting relates to turnover intention reports conflicting findings. Specifically, Esteves and Lopes (2016) found that job crafters have a low level of turnover intention, and no mediators are reported there. However, a meta-analysis showed that job crafting, as an overall construct, is not significantly related to turnover intention (Rudolph et al., 2017). We therefore speculate that there might be a certain offsetting effect in the overall relation. Therefore, identifying the paths that may have positive and negative impacts on the job crafting-turnover intention association is now both timely and necessary.

In view of the above points, we tend to determine the essential factors that can explain the negative link between job crafting and turnover intention and then to see whether the direct relation could be reversed after controlling the intermediate mechanism. We address that organizational instrumentality is the key mediating factor in facilitating this negative indirect relation. Organizational instrumentality refers to employees’ perception that the organization will be instrumental in helping them reach personal goals (Fleishman et al., 1991; Cardador et al., 2011), which is in accordance with the core connotation of goal facilitation theory addressing the motivated effect of goals (Fitzsimons and Shah, 2008). We choose organizational instrumentality as the mediator because, on the one hand, individuals with clear goals tend to make a comprehensive evaluation of the current organization before they decide to stay or leave, and organizational instrumentality is such a kind of overall appraisal about the utility of the organization for their goals (Cardador et al., 2011). Although some positive results of job crafting—for example, person-job fit, job satisfaction—may also negatively predict turnover, these work-related variables are employees’ evaluation of a certain facet of the organization rather than the overall appraisal. Thus, choosing organizational instrumentality as a mediator may help to understand the essential mechanism between job crafting and turnover intention. On the other hand, given the goal-oriented characteristics of job crafting (Wrzesniewski and Dutton, 2001), goal facilitation theory provides a plausible and overarching lens for explaining how individuals with clear and important goals (i.e., job crafters) approach and utilize a particular environment to shape their evaluation of the environment (i.e., organizational instrumentality) and then trigger the consequent behaviors toward the environment (i.e., turnover intention) (Fitzsimons and Shah, 2008).

Leaders are an important prerequisite for goal facilitation (Antonakis and House, 2014). Theoretically, given that goal facilitation theory highlights the supportive factors that facilitate an employee’s goal fulfillments, as a proximal influential factor, supervisory behaviors are treated as providing support or limiting resources for the purpose of assisting in followers’ goal attainment (Fleishman et al., 1991; Morgeson et al., 2010). Job crafting is a process full of obstacles, risks and unexpected problems (Wrzesniewski and Dutton, 2001); thus, whether job crafters can obtain support from leaders may either facilitate or impede the realization of crafting aims. As a relational leadership approach, inclusive leadership represents leaders who are open and accessible to subordinates (Carmeli et al., 2010) and cultivate a context where individuals are given more tolerance, trust and assistance when taking risks or making mistakes during crafting the job, which is more particular in the Chinese *guanxi* context. Therefore, these job crafters feel safer (Carmeli et al., 2010) in looking to their originations for opportunities and resources to fulfill their goals; accordingly, organizational instrumentality can be brought into full play, which further affects job crafters’ evaluation and behavioral intention toward organizations. Therefore, we address that inclusive leadership may activate the benefits of job crafting for organizational instrumentality and then decrease turnover intention.

Finally, we are also concerned with the direct positive effect that job crafting exerts on turnover intention. Tims and Bakker (2010) show that job misfit is a main reason for employees to craft jobs. Job crafters, as being considered to have a trait of proactivity (e.g., Tims and Bakker, 2010; Tims et al., 2013), are also willing to make changes to undesirable work by actively pursuing all kinds of possibilities outside the organization that could promote career growth, especially when they feel limited or fail in job crafting due to various constraints in their working settings (e.g., Berg et al., 2010). Therefore, we suggest that job crafters will not passively adapt to undesirable work but tend to leave their current organizations when we control the increased instrumentality of organizations for themselves and when controlling the inclusive leadership under which employees can perceive that the leader welcomes and accepts their diverse job-crafting goals (Hantula, 2009; Carmeli et al., 2010).

**FIGURE 1:**
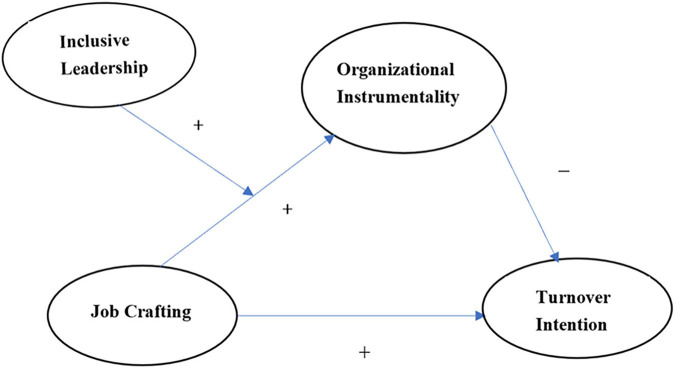
The hypothesized model.

Empirically, we delineated and tested a latent moderated mediation model (see the hypothesized model in Figure 1) with a sample of 218 knowledge employees of high-tech companies to clarify how job crafting relates to turnover intention. Our study adds to the promising idea on this relationship, which has received much less empirical attention in previous studies (for example Esteves and Lopes, 2016; Rudolph et al., 2017). Specifically, the present study aims to make three main contributions to the current literature. First, on the basis of goal facilitation theory, we bridge the theoretical gap in the underlying mechanism by explaining how job crafting may decrease turnover intention by facilitating organizational instrumentality. Second, we identify inclusive leadership operation as a facilitating condition that contributes to an enhanced understanding of the social environment conditions under which goal facilitation theory fully works and helps practitioners develop and use inclusive leadership interventions to decrease the turnover intention of job crafters in contemporary organizations. Finally, by addressing how job crafting positively relates to turnover intention, our study provides new insights for understanding the double-edged effects that job crafting plays on turnover intention.

## Theoretical Background and Hypotheses Development

### Conceptualization of Job Crafting

Employees are not passive recipients of traditional top-down job design but rather are positive in creating their work experiences by proactively modifying their jobs (Bell and Staw, 1989). Wrzesniewski and Dutton (2001) defined job crafting as “the physical and cognitive changes employees make in the task or relational boundaries of their work” (p.179). In the original framework of job crafting (Wrzesniewski and Dutton, 2001), individuals are motivated to craft job boundaries in three ways: task crafting, relational crafting and cognitive crafting. Both task crafting and relational crafting are behavioral changes conducted by individuals while performing their jobs, whereas cognitive crafting involves employees’ recognition of their jobs. Because cognitive changes are more strongly related to individuals’ inner desires, these changes are not easy to make and do not involve actual changes in a job (Demerouti, 2014). Thus, job crafting mainly focuses on how individuals act to change the physical and relational boundaries of the jobs in ways that better fit their motivation, skills, and interests (Ghitulescu, 2007), and previous studies have taken job crafting as a broad construct with two dimensions (physical crafting and relational crafting) and have shown good applicability in empirical studies (Laurence, 2010; Lu et al., 2014).

### Job Crafting, Organizational Instrumentality and Turnover Intention

From the insights of goal facilitation theory, we advanced the understanding of logical processes by which employees’ job crafting behaviors decrease their turnover intention through organizational instrumentality.

#### The Relation Between Job Crafting and Organizational Instrumentality

Goal facilitation theory places much emphasis on the accelerating effect of personal meaningful goals (Fitzsimons and Shah, 2008). Specifically, individuals motivated by the goals will automatically seek social environments that may help in advancing these goals, and in that regard, the particular environment may be perceived instrumental to personal goal achieving (Fitzsimons and Shah, 2008). Previous experimental studies also confirmed that the sense of effort with goals makes people have more perception of instrumentality of a particular environment during goal pursuit (Labroo and Kim, 2009). Career scholars have consistently captured this perception with the term organizational instrumentality (Cardador et al., 2011). For working adults, organizations are the most pivotal social environment where they could have access to all kinds of resources. Job crafting is a kind of goal-driven behavior that aims to achieve a better fit between one’s job and their preferences (Tims and Bakker, 2010; Bakker et al., 2012). In light of the theory, job crafters will actively overcome various obstacles to using the organizational environment. In fact, job crafting is also considered to be a process of searching, utilizing and increasing resources in the current organization (Tims and Bakker, 2010; Bakker et al., 2012). Specifically, for task crafting, individuals always keep a weather eye on and try to obtain the resources in the organization, such as attending possible project opportunities that can help improve their ability and experience, seeking available equipment, technology or support to improve work efficiency (Laurence, 2010); for relational crafting, individuals actively identify and reshape instrumental ties with important others within the organization. On the basis of the theory (Fitzsimons and Shah, 2008), individuals’ important and meaningful goals can be advanced in the crafting efforts of utilizing organizations, which in turn will improve employees’ evaluation of organizational instrumentality. As a supplement, Labroo and Kim (2009) addressed the correspondence between the feeling of instrumentality and the actual efforts during goal pursuit; that is, the more effort they make, the more instrumentality they will feel. Taken together, we propose that:

Hypothesis 1: Job crafting is positively related to organizational instrumentality.

#### The Relation Between Organizational Instrumentality and Turnover Intention

Additionally, goal facilitation theory suggests that individuals who have important and active goals have a greater tendency to evaluate instrumental others positively, and then they will be more ready to approach them (Fitzsimons and Shah, 2008). Because organizational instrumentality provides the necessary resources and supports employees’ goal achievement (Cardador et al., 2011), employees will obtain a sense of satisfaction with work and engage more in their work (Xanthopoulou et al., 2009; Tims et al., 2013; Xie et al., 2017). Moreover, when perceiving the organization as instrumental, individuals may keep investing more efforts in getting more resources from the organization (Benson, 2006). For example, they are more likely to participate in activities in their organizations because of increased membership (Aryee and Chay, 2001). In that regard, employees may have less turnover intention. We propose that:

Hypothesis 2: Organizational instrumentality is negatively related to turnover intention.

#### Organizational Instrumentality as a Mediator

Goal facilitation theory provides an overarching view for explaining how individuals with clear and active goals approach the instrumental environment to shape their evaluation of the person-organization relationship (Fitzsimons and Shah, 2008). A previous study confirmed that individuals with goals generate their positive or negative behaviors and attitudes toward the organization through the evaluation of organizational instrumentality (Xie et al., 2017). From that, instrumentality helps to form individuals’ evaluation of person-organization relationships; as such, the more instrumentality they feel, the stronger they link to the organization, which acts as the most proximal predictor of turnover. Drawing on the theory, *Hypothesis 1* and *Hypothesis 2* explained why job crafters are more likely to perceive organizational instrumentality and how this perception further affects their turnover intention. Taken together, we propose that:

Hypothesis 3: Organizational instrumentality mediates the relationship between employee job crafting and turnover intention.

### Inclusive Leadership as a Moderator

Goal facilitation theory highlights that individuals will perceive the environment more instrumental when they have got closer to their goals in such an environment (Fitzsimons and Shah, 2008). Leaders are the actual distributor and controller of work resources (Fleishman et al., 1991; Morgeson et al., 2010) and are undoubtedly one of the most significant others for goal realization. Along this line of theorizing, given that the goal behavior of employees at work is often implemented within a particular organization, we propose that the instrumentality of leaders that can facilitate goal attainment could be diffused. In particular, employees under the condition of instrumental leaders will evaluate the whole organization more positively during goal pursuit.

Some scholars have claimed that since employees may encounter difficulties and all kinds of constraints in crafting their job boundaries, job crafting is characterized as a process of the continuous consumption of personal energy (Demerouti et al., 2015). In that regard, supportive leadership matters in the way that it may either facilitate or impede the realization of crafting aims. Evidence from prior studies has shown that leaders who employ desirable supervision may shape the results of job crafting (Wang et al., 2016). Representing the relation between a leader and subordinate based on respect, response, and responsibility (Hantula, 2009), inclusive leadership is characterized as a leader’s appreciation and recognition of his or her followers’ contribution (Nembhard and Edmondson, 2006) and can be conceptualized as “leaders who exhibit openness, accessibility, and availability in their interactions with followers” (Carmeli et al., 2010).

Previous research has indicated that supervisory behaviors characterized as supportive and developmental can help make a favorable context for followers to achieve goals by job crafting (Leana et al., 2009). From this point, by paying attention to employees’ personal needs, the inclusive leadership approach could be propitious to the aims of employee job crafting. Specifically, the openness of inclusive leadership recognizes individual differences with an open mind and recognizes the diversity of subordinates’ personal goals (Carmeli et al., 2010). At the same time, the accessibility and availability of inclusive leaders will also let job crafters feel more confident and be more driven to overcome barriers. Taken together, in the inclusive context, job crafters could feel safer and bolder to obtain all the possible resources within the current organization, such as funding, equipment, project opportunities or social connections (Nishii and Mayer, 2009; Carmeli et al., 2010; Hirak et al., 2012). Consequently, employees are more likely to believe their crafting aims can be facilitated in the current organization because of the inclusiveness of their leaders, which accordingly is succeeded by the higher perception of organizational instrumentality (Fitzsimons and Shah, 2008). As discussed earlier, with the higher perception of organizational instrumentality, employees will be more willing to invest in the current organization to approach their goals, which in turn improves their organizational membership and reduces turnover intention. Therefore, under more inclusive leadership, the indirect relation between job crafting and turnover intention will be enhanced through instrumentality. In contrast, the mediating effect of organizational instrumentality is weaker. Thus, we propose that:

Hypothesis 4: Inclusive leadership plays a moderating role in the relationship between job crafting and organizational instrumentality such that with more inclusive leadership, the relationship is stronger.
Hypothesis 5: Inclusive leadership plays a moderating role in the mediated relationship between employee job crafting and turnover intention through organizational instrumentality in such a way that with more inclusive leadership, the relationship is stronger.

### How Job Crafting Relates to Turnover Intention Directly

Job crafting often occurs when employees perceive some dissatisfaction or misfit at work because it is thought to be a means of solving problems in the current job that formal organizational design cannot solve (Wrzesniewski and Dutton, 2001). For example, Tims and Bakker (2010) proposed that P-J misfit is a primary cause of job crafting. We address that the condition of dissatisfaction and misfit will not disappear promptly with the process of job crafting. However, job crafting processes may not always be smooth because of many possible constraints, such as misalignments between crafting behaviors and organizational expectations (e.g., Oldham and Hackman, 2010), conflicting role sets (Dierdorff and Jensen, 2018), misunderstandings of crafting behaviors from peers and leaders (Lyons, 2008), and other limited crafting resources (e.g., time and autonomy) (e.g., Berg et al., 2010). Similarly, a qualitative study by Berg et al. (2010) revealed that job crafting relates to increased job strain and intermittent feelings of regret. As such, job crafters may turn to other alternatives when they cannot effectively overcome the pressure and obstacles in job crafting.

Turnover intention is a kind of coping strategy when employees are under unsatisfactory work conditions (Avanzi et al., 2014). Job crafters are generally believed to have the trait of proactivity (e.g., Tims and Bakker, 2010; Bakker et al., 2012), which has been found to be positively associated with certain critical antecedents of actual turnover, such as career self-efficacy and job search self-efficacy (Fuller and Marler, 2009). Thus, job crafters can have tendencies to take initiative to change their current situations by actively pursuing all kinds of possible opportunities and alternatives outside the organization for the purpose of career advancement. Several scholars have found that turnover intention is triggered when employees perceive themselves as having more opportunities in the labor market (e.g., Benson et al., 2004; De Cuyper et al., 2011; Nelissen et al., 2017). We argue that job crafters are more likely to find alternative opportunities outside the organization with their continuously enhanced competencies in crafting tasks (Lyons, 2008; Petrou et al., 2012) and with vital talent market information obtained from instrumental ties in relational crafting (e.g., Bakker et al., 2012).

In summary, considering that job crafting stems from dissatisfaction with the current job and insurmountable obstacles that job crafters may encounter, we predict that job crafters will not passively adapt to undesirable work but tend to leave their current organizations when the conditions for changing work are met and satisfactory external opportunities appear. However, it should be noted that this is on the premise of ignoring the roles of instrumental and inclusive leadership of the organization. Thus, we propose that:

Hypothesis 6: The relation between job crafting and turnover intention is positive when controlling the roles of organizational instrumentality and inclusive leadership.

## Materials and Methods

### Sample and Procedure

The survey was spread out randomly by using either paper copies or the online way and all subjects voluntarily participated the survey. Finally, we recruited 218 employees from ten high-tech companies in Beijing and Shenzhen as participants. These high-tech companies were mainly from the internet finance, communication, and high-tech energy industries. All questionnaires were filled out by the employees. The sample included a variety of occupations, including technology and development, market, product operation, business development, administrative personnel and other functional areas. Considering the nature of the research variables, all variables in the study were by employees themselves. To avoid homology bias, we collected time lagged data, with an interval of 30 days. In the first-round survey, we distributed 318 questionnaires and obtained 268 valid responses. After 30 days, the second round of data collection was conducted with the participants, and 218 valid copies were obtained. Variables measured at the first time point were job crafting and the inclusive leadership style of employees’ direct supervisors. The variables measured at the second time point were organizational instrumentality and turnover intention. Among the participants, 142 (65%) subordinates were male, and 76 (35%) were female. Participants had an average age of 30.1 years (SD = 7.3). On average, participants had 6.25 years (SD = 4.5) of work experience in the company. Four (1.8%) respondents had an education level of high school or below, 12 participants (5.5%) had a high school education level, 45 (20.7%) held associate degrees, 136 (62.7%) held bachelor’s degrees, and 20 (9.2%) held master’s degrees or above.

### Measurement

#### Job Crafting

Employee job crafting was assessed by the expansion-oriented job-crafting scale with 18 items (Laurence, 2010) to evaluate the degree of frequency that employees crafted the job. This scale has two subdimensions, including physical and relational. Respondents valued each item of the scale on a 5-point Likert scale, from “not at all” to “very much so.” Sample items for each dimension were “I have taken steps to increase the challenges I am facing in my job” and “I have taken steps to increase the extent to which I deal with other people in my job.” The scale’s internal consistency was 0.92.

#### Organizational Instrumentality

Organizational instrumentality in our study was measured by the four items with one dimension, developed by Cardador et al. (2011). Respondents rated each item on a 6-point Likert scale, from “strongly disagree” to “strongly agree.” One sample item was “Working in my organization helps me to achieve my personal goals.” The scale’s internal consistency was 0.89.

#### Inclusive Leadership

Employees rated their direct supervisors’ inclusive leadership with inclusive leadership in three dimensions, nine items in total, developed by Carmeli et al. (2010). Subdimensions of the scale include openness, availability and accessibility, and each dimension contained three items. Respondents valued each item on a 6-point Likert scale, from “strongly disagree” to “strongly agree.” Sample items for each dimension were “My supervisor is open to listening to some new ideas,” “My supervisor is available for consultation on problems,” and “My supervisor is accessible for discussing emerging problems.” The scale’s internal consistency was 0.91.

#### Turnover Intention

Employees rated their turnover intention with three items, combined into one dimension and developed by Konovsky and Cropanzano (1991). Participants valued from “totally disagree” to “totally agree” on a 5-point Likert scale. A sample item was “I often think about leaving this organization.” The total scale’s internal consistency was 0.87.

#### Control Variables

Certain demographic variables have previously been found to affect turnover intention (Chang et al., 2013; Gyensare, 2016). To make our model testing more accurate, we included three demographic variables as potential control variables in this study, all of which were assessed at Time 1. We controlled for gender (0 = male, 1 = female), education (1 = high school level or below, 2 = high school level, 3 = associate degree, 4 = bachelor’s degree, 5 = master’s degree or above) and organizational tenure.

## Results

### Confirmatory Factor Analysis and Descriptive Results

To test the distinguishing validity of our model, we conducted confirmatory factor analysis (CFA) through Mplus 7.0 with the maximum likelihood estimation. Considering our sample size, we used item parcels with an internal-consistency approach to make the analysis tractable. Job crafting was modeled as a latent factor with five indicators (i.e., improving task function, seeking challenges and opportunities, seeking autonomy, expanding connections, and improving the qualities of connections). Inclusive leadership was modeled with three indicators (i.e., openness, availability, and accessibility). For the organizational instrument, turnover intention, which was measured with no more than four items, we kept their original items. We compared our hypothesized model with alternative models. The CFA results indicated that the four-factor model distinguishing among job crafting, inclusive leadership, organizational instruments and turnover intention was significantly better than the other three models.

Table 1 shows the results of the descriptive analysis as well as the correlations of the variables. The results showed that job crafting was positively related to organizational instrumentality (*r* = 0.38, *p* < 0.01) and inclusive leadership (*r* = 0.39, *p* < 0.01) and weakly negatively related to turnover intention (*r* = −0.14, *p* < 0.05).

**TABLE 1:** Descriptive statistics, reliability coefficients, and intercorrelations among variables.

	**Mean**	**SD**	**1**	**2**	**3**	**4**	**5**	**6**	**7**
1. Gender	1.35	0.48	–						
2. Education	3.72	0.78	0.15^*^						
3. Tenure	6.25	4.50	0.16^*^	−0.16^*^					
4. Job Crafting	2.33	0.69	−0.06	0.08	−0.05	(0.91)			
5. Organizational Instrumentality	3.73	0.75	0.13	0.14^*^	−0.20^*^	0.38^**^	(0.89)		
6. Inclusive Leadership	4.89	0.86	−0.02	0.15^*^	−0.28^*^	0.39^**^	0.52^**^	(0.91)	
7. Turnover Intention	2.41	0.94	−0.10	−0.12	0.15^*^	−0.14^*^	−0.49^**^	−0.35^**^	(0.87)

*Reliability coefficients appear in brackets on the diagonal.*

*p < 0.05 and **p < 0.01.

### Testing Hypotheses

Following the suggestion of Cheung and Lau (2017), the moderated mediation model was tested using LMS equations. In contrast to the popularly used regression method with biased estimates of regression coefficients, the LMS equation approach corrects for measurement errors to produce more accurate parameter estimates and confidence intervals (CIs) when estimating latent interaction effects. We followed the approach of previous studies, and a 3-step procedure was conducted using Mplus 7.0. with maximum likelihood estimation.

First, we assessed the overall model fit of the moderated mediation model. Because the usual fit indices are not provided when estimating the latent interaction, we estimated a model from which the latent interaction term was excluded to obtain the conventional fit indices. The model without the latent interaction showed a good fit [χ^2^ (111) = 288.38; TLI = 0.92, CFI = 0.90; RMSEA = 0.08, SRMR = 0.07].

**FIGURE 2:**
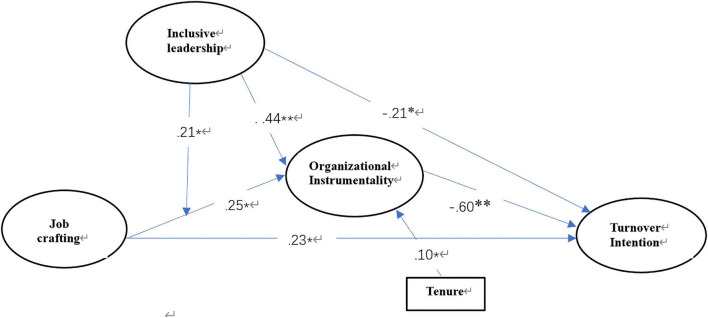
Unstandardized path estimates of the final model. ^∗^*p* < 0.05 and ^∗∗^*p* < 0.01.

Second, the model in Figure 2 with a latent interaction between job crafting and inclusive leadership, and the path from the interaction to organizational instrumentality was estimated. In consideration of the abnormal distribution of the mediating effect and interaction term, we used bootstrap estimates in which two thousand bootstrap samples were generated and constructed bias-corrected bootstrap CIs to test each estimated parameter in the current analysis. First, we tested the proposed model, which was the moderated mediation model with direct effects, and a summary of the results, including all the unstandardized path estimates and CIs, is presented in Figure 2 and Table 2.

**TABLE 2:** Path coefficients for the moderated mediation models.

	**Organizational instrueDespite substantial reseamntality**	**Turnover intention**
Job crafting	0.247^*^^a^ [0.105, 0.390]^b^	0.231^*^ [0.105, 0.390]
Inclusive leadership	0.442^*^^*^ [0.318, 0.566]	−0.214^*^ [−0.387, −0.024]
Interaction: job crafting × inclusive leadership	0.207^*^ [0.081, 0.027]	___
Organizational instrumentality	___	−0.600^*^^*^ [−0.791, −0.382]
Education	−0.009 [−0.020, 0.000]	0.001 [0.013, 0.015]
Tenure	0.103^*^ [0.003, 0.227]	−0.027 [−0.161, 0.108]
*R* ^2^	0.447	0.417

*^*a*^Unstandardized path estimates.*

*^*b*^95 percent bias-corrected confidence intervals.*

^*^p < 0.05 and ^*^^*^p < 0.01.

As shown in Table 2, job crafting positively impacts organizational instrumentality (*b* = 0.25, *p* < 0.05). Organizational instrumentality has a significantly negative effect on turnover intention (*b* = −0.60, *p* < 0.01). Job crafting’s indirect effect on turnover intention is the multiplication of the above two path coefficients. The results show that the CI (95%) of the indirect effect does not overlap zero, which indicates that job crafting has a statistically significant indirect effect on turnover through organizational instrumentality [estimate = 0.15, *p* < 0.05, bias-corrected CI (−0.30, −0.026)]. Hypotheses 1, 2, and 3 are fully supported. Moreover, we note that the direct path coefficients between job crafting and turnover intention are significantly positive (*b* = 0.23, *p* < 0.01), and Hypothesis 6 is supported.

**FIGURE 3:**
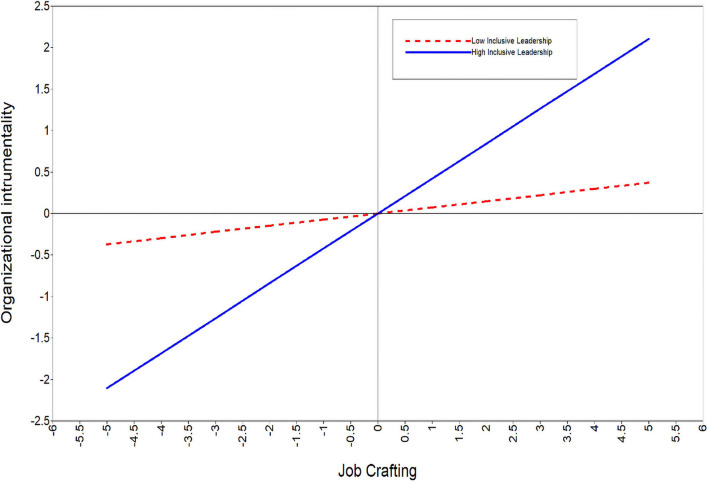
The moderation effect of inclusive leadership on the relationship between job crafting and organizational instrumentality.

Regarding the moderation of inclusiveness between job crafting and organizational instrumentality (the simple moderation in the first stage), the interaction of job crafting and inclusive leadership significantly predicts organizational instrumentality [*b* = 0.21, *p* < 0.05, bias-corrected CI (0.08, 0.03)]. When inclusive leadership is high, the simple slope is very significant (*b* = 0.42, *p* < 0.01; see the solid line in Figure 3), suggesting that job crafting relates to organizational instrumentality more closely. When inclusive leadership is low, the simple slope is not significant (*b* = 0.07, *p* > 0.05; see the dashed line in Figure 3), suggesting that job crafting has no significant effect on organizational instrumentality. Hypothesis 4 is fully supported.

To further confirm whether inclusive leadership has a moderating effect on the direct effect, as a supplementary analysis, we tested an alternative model where a path from the interaction between job crafting and inclusive leadership to turnover intention was added. The path between the interaction term and turnover intention is non-significant [*b* = −0.02, *p* > 0.10, 90% bias-corrected CI (−0.17, 0.14)]. The results show that inclusive leadership dose not play a moderating role in the direct effect between job crafting and turnover intention. Hypothesis 6 is fully supported. Since gender is not related to all the key variables in the correlation analysis, we removed it from the structural equation model. Regarding controlled demographic variables, we found that only tenure was positively related to organizational instrumentality (*b* = 0.10, *p* < 0.05).

**FIGURE 4:**
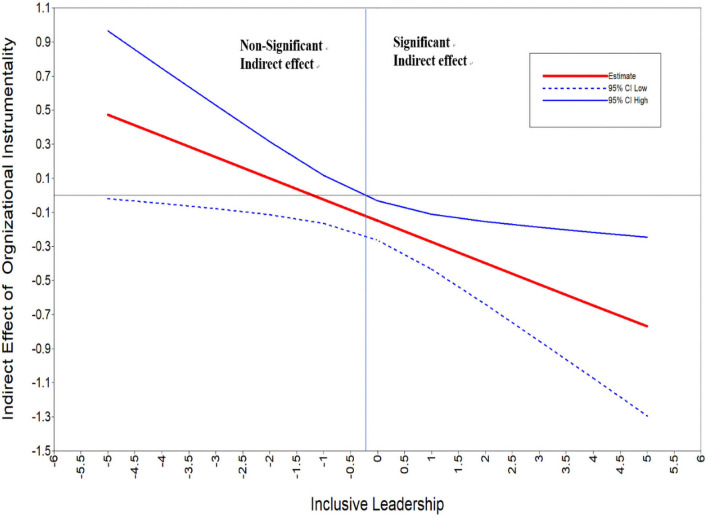
The moderation effect of job crafting on turnover intention through organizational instrumentality.

Third, following the suggestion by Cheung and Lau (2017), we examined the conditional indirect effect by analyzing the magnitude and significance of the indirect effect that job crafting played on turnover intention *via* organizational instrumentality at various levels of inclusive leadership. The analysis results indicated that at a high level of inclusive leadership (+1 standard deviation), the indirect effect that job crafting played on turnover intention was significantly negative [estimate = −0.25, *p* < 0.01, bias-corrected CI (−0.40, −0.10)], and at a zero level of inclusive leadership (mean), job crafting had a significant indirect effect on turnover intention [estimate = −0.15, *p* < 0.05, bias-corrected CI (−0.26, −0.04)], while at a low level of inclusive leadership (−1 standard deviation), job crafting had no significant indirect effect on turnover intention (estimate = −0.04, *p* > 0.10, bias-corrected CI [−0.18, 0.07]. We plotted the conditional indirect effect among the variables (see Figure 4). As shown in Figure 4, higher inclusive leadership was negatively related to a stronger indirect effect that job crafting played on turnover intention through organizational instrumentality. Only when inclusive leadership was at levels more than 0.2 standard deviations below the mean was the indirect effect significant. Hypothesis 5 is fully supported.

## Discussion

The current study, which sought to clarify how job crafting complications were associated with turnover intention, examined this relation by identifying organizational instrumentality as a mediator and inclusive leadership as a moderator. On the one hand, the findings indicated that employees’ job crafting was positively related to their perception of organizational instrumentality, which led to decreased turnover intention, thereby suggesting that organizational instrumentality works as an intervention in the negative relationship between job crafting and turnover intention. Moreover, we also found that inclusive leadership not only positively moderated the path from job crafting to organizational instrumentality but also moderated the entire mediational relationship. The findings of the current research indicate that the benefits of job crafting are strengthened when inclusive leadership is high. On the other hand, we found that job crafting positively and directly impacted turnover intention; that is, after controlling for organizational instrumentality and inclusive leadership, we found that job crafting was positively related to turnover intention.

### Theoretical Implications

This research has several contributions to studies on job crafting and turnover intention. First, we examined the complex effect that individual job crafting played on turnover intention. Specifically, taking both the positive and negative effects that job crafting played on turnover intention into consideration, we enriched the current understanding of job crafting by enlightening the dysfunctional effects of job crafting in the workplace. From the perspective of the negative influence of job crafting on turnover intention, we applied goal facilitation theory to provide new evidence to clarify the mechanisms by which job crafting decreases turnover intention. The findings specifically highlight that higher job crafting of employees will lead to higher perceived organizational instrumentality, increasing the likelihood that employees are stimulated and thereby decreasing their turnover intention. By employing the theoretical framework of goal facilitation theory, we found that when employees craft their jobs, they prefer to view their organizations as useful instruments to help them realize their goal of customizing jobs to their own specifications. This result is consistent with broad insight into the goal perspective of job crafting behaviors (Wrzesniewski and Dutton, 2001; Van den Heuvel et al., 2015). That is, job crafting is a goal-oriented behavior. Employees with job crafting have a clear goal of making their tasks match their own preferences. Furthermore, given that job crafting requires job resources, we found that organizational instrumentality can be instrumental because it can provide relevant resources toward successful job crafting. Accordingly, the fulfillment of job crafting boosts people’s attachment to the organization and decreases their turnover intention.

From the perspective of the positive influence that job crafting played on turnover intention, we proposed and found that job crafting relates to turnover intention directly and positively after controlling for the mediator (i.e., organizational instrumentality) and moderator (i.e., inclusive leadership). That is, consistent with certain previous studies (e.g., Demerouti et al., 2015), the current study included estimation of the potential dark side of job crafting. Our results highlighted the “opportunity and resources” for job crafters. Specifically, employees craft their jobs due to dissatisfaction with their work; therefore, they tend to look for additional responsibilities and challenges (Petrou et al., 2012). According to signaling theory, individuals may view such extra responsibilities and developments as powerful signals of their own abilities to prospective employers (Spence, 1974; Acemoglu and Pischke, 1999) and may therefore perceive themselves as having a stronger position in the external labor market, which could potentially positively influence their turnover intention (Nelissen et al., 2017). In this vein, our findings contribute to the proactive literature by highlighting that job crafters who are characterized as proactive employees can display proactive behaviors—i.e., adapting to changes in the work situation and changing aspects of their work environment themselves—to achieve desirable outcomes.

Moreover, we extend the limited but growing research that acknowledges a “dark side” of job crafting. That is, the findings in our study explicitly show the double-edged nature of job crafting, which is also indicated by the weak but negative correlation between job crafting and turnover intention (*r* = −0.14, *p* < 0.05; Table 1). In this vein, we address the mixed findings of the nature of job crafting in the workplace by showing that job crafting may also negatively relate to turnover intention. Specifically, with respect to the positive effect, the more employees craft their jobs, the more opportunities and resources they may obtain to find more satisfactory jobs, which demonstrates career orientation from the outsider perspective. In contrast, regarding a negative influence, employees’ job crafting may cause them to rely more on their organizations to make changes and subsequently lead them to report lower levels of turnover intention, which highlights career orientation from the insider perspective. This phenomenon indicates that organizational instrumentality can diminish the positive effect that job crafting plays on turnover intention. In this regard, future research that would enrich the literature on job crafting by examining dysfunction in job crafting among employees at the workplace is highly encouraged.

In revealing the two sides, the paper focused on how job crafting reduces turnover intention (indirect path). The main reason lies in that organization and manager would prefer to knowing what really makes job crafters stay. In this regard, we tend to figure out the most essential factors that can explain the negative link between job crafting and turnover intention, and then to detect whether the direct relation could be reversed after controlling the intermediate mechanism. Our explorations suggested managers that the indirect path should be highly emphasized, because the higher the level of job crafting, the more likely employees are to leave. This also reflected the unique value of choosing organizational instrumental and inclusive leadership as the mediator and the moderator, respectively.

Furthermore, our results regarding the moderating role of inclusive leadership extend the current understanding of goal facilitation theory. Specifically, although conceptual research has consistently highlighted leadership as a prerequisite for goal facilitation, limited empirical studies have been conducted to examine this proposition in the domain of proactivity literature. In our study, we identified a specific leadership style, inclusive leadership, to clarify that the impacts of employee job crafting with respect to increasing organizational instrumentality and then decreasing turnover intention can be strengthened by a high level of inclusive leadership. In this vein, we enrich the current theoretical understanding of leaders as an important prerequisite for goal facilitation (Antonakis and House, 2014). Extending previous research primarily suggesting that leadership styles may influence employee job crafting (Breevaart et al., 2016; Wang et al., 2017), our study further empirically identified the boundary condition of inclusive leadership in the job crafting literature. That is, when leaders enact behaviors that are inclusionary for their followers, these followers’ job crafting behaviors are more likely to reduce turnover intention. Since inclusive leadership highlights the value of uniqueness (Randel et al., 2018), it not only provides employees with more job autonomy but also creates a more psychologically safe environment where employees are allowed to show job crafting behaviors to pursue their own goals. Specifically, employees working with a more inclusive leader can feel more belongings, respected, and less stressed (e.g., Ashikali et al., 2021); therefore, they are more likely to be proactive by crafting their jobs in the workplace. As a result, they tend to perceive the instrumentality of their inclusive social environment in fulfilling their goal of crafting jobs toward decreasing their turnover intention. We also found inclusive leadership failed to simply moderate the relationship between job crafting and turnover intention, which indicates that the moderating role of inclusive leadership can only be played in the indirect path though promoting the job crafters’ perception of organizational instrumentality.

### Practical Implications

This study has certain practical implications. First, our findings demonstrate the fact that employees engaging in job crafting should be highly valued and encouraged. Thus, employees should develop their own mindset to actively use their job demands and resources. To attract proactive employees, organizations could benefit greatly from providing job crafting opportunities to employees as well as giving them specific training that aims to develop their personalized job crafting goals. Supervisors should also show their tolerance and let the subordinates do their daily job in their own ways. When employees are self-determined, they could make choices freely on tasks that they truly enjoy, resulting in lower levels of turnover intention. In addition, job crafting, a bottom-up job redesign strategy, has been revealed to be meaningful to employees. Therefore, for managers to improve the work environment, adding job crafting as an initiative in a top-down way has been enlightened.

Furthermore, given the research findings on the double-edged nature of job crafting among employees, it would be important for job crafters to take advantage of organizational instrumentality. In this way, they can decrease their turnover intention by putting more effort into the workplace. Specifically, we encourage modern organizations to protect and maintain staff’s perceived organizational instrumentality. For example, managers are encouraged to remove obstacles that hinder the accomplishment of followers’ work-related goals, thereby increasing perceptions of organizational instrumentality.

To manage individuals with job crafting behaviors, organizations should protect and maintain these workers’ perceptions regarding organizational instrumentality. In this manner, the negative effect that job crafting played on turnover intention can be strengthened, and the possibility of job crafting having a positive effect on turnover intention can be avoided. We encourage managers to reduce barriers that impede subordinates’ work goal achievement and further increase the sense of organizational instrumentality; for the HR department, we suggest that managers create psychological conditions that are similar to high levels of job crafting behaviors to let them feel the work is personally fulfilling.

Given the results about inclusive leadership, leaders should develop and enact an inclusive leadership style to facilitate employees’ job crafting and decrease employees’ turnover intention. Specifically, organizations should provide training courses to help managers be more inclusive when supervising their subordinates, such as by creating a participative environment.

### Limitations and Future Research

Limitations cannot be ignored. First, although we used a time-lagged research design, the possibility of a causality problem cannot be entirely excluded because organizational instrumentality and turnover intention were both measured at the same time (Time 2). That is, although our proposition, consistent with previous studies showing that organizational instrumentality leads to employees’ desirable outcomes (e.g., organizational attachment) (Haworth and Levy, 2001), claims that organizational instrumentality can decrease employees’ turnover intention, it is possible that employees’ turnover intention may affect their perception of organizational instrumentality because turnover intention may reduce employees’ work engagement and their organizational citizenship behavior (Xiong and Wen, 2020). Therefore, to replicate our results, a longitudinal research design is suggested in future research to establish causality. Second, the sample for our research was extremely specific to the high-tech industry in China, limiting the validity and generalizability of our findings. Validity and generalizability could be increased by testing our model with a sample from a different industry (e.g., a service industry) in a different Asian country. Moreover, we collected data from only one source (employees). Although the results show that CMB is not a problem in our study, future research involving the collection of data from multiple sources is still encouraged. Finally, although we proposed a moderated mediation model to test the dysfunctional effect of job crafting on turnover intention, future research to explore why (*via* which intervening mechanism) and when (under which boundary conditions) job crafting is positively related to employees’ turnover intention remains recommended.

## Data Availability Statement

The raw data supporting the conclusions of this article will be made available by the authors, without undue reservation.

## Ethics Statement

The studies involving human participants were reviewed and approved by Southwest University of Political Science and Law. Written informed consent for participation was not required for this study in accordance with the national legislation and the institutional requirements.

## Author Contributions

XX and WC: conceptualization and funding acquisition. XX and XG: methodology. XX: formal analysis, resources, and project administration. WC: investigation. XG and TL: data curation. XX, WC, and XG: writing—original draft preparation. WC, XG, and TL: writing—review and editing. All authors have read and agreed to the published version of the manuscript.

## Conflict of Interest

The authors declare that the research was conducted in the absence of any commercial or financial relationships that could be construed as a potential conflict of interest.

## Publisher’s Note

All claims expressed in this article are solely those of the authors and do not necessarily represent those of their affiliated organizations, or those of the publisher, the editors and the reviewers. Any product that may be evaluated in this article, or claim that may be made by its manufacturer, is not guaranteed or endorsed by the publisher.
